# Acceptance and utilization of web-based self-help for caregivers of children with externalizing disorders

**DOI:** 10.1186/s13034-024-00724-0

**Published:** 2024-03-25

**Authors:** Laura Wähnke, Julia Plück, Mikel Bodden, Angela Ernst, Marie-Theres Klemp, Judith Mühlenmeister, Manfred Döpfner

**Affiliations:** 1 School for Child and Adolescent Cognitive Behavior Therapy (AKiP), Faculty of Medicine, University Hospital Cologne, University of Cologne, Cologne, Germany; 2 Institute of Medical Statistics and Computational Biology, Faculty of Medicine and University Hospital Cologne, University of Cologne, Cologne, Germany; 3 Department of Child and Adolescent Psychiatry, Psychosomatics and Psychotherapy, Faculty of Medicine and University Hospital Cologne, University of Cologne, Cologne, Germany

**Keywords:** Web-based self-help, Parent management training, Utilization, Acceptance, Externalizing symptoms, Children, Predictors

## Abstract

**Background:**

Web-based self-help interventions for parents of children with ADHD and other externalizing disorders have been proven to be effective. In order to recommend individualized and optimized interventions, a better understanding of the acceptance and utilization of this innovative treatment approach is needed. Previous research has frequently employed subjective reports of utilization, but the validity of these studies may be limited.

**Methods:**

Data from the German WASH study were used. Participants (*n* = 276) were randomly assigned to the intervention condition (a) web-based self-help or (b) web-based self-help with optional telephone-based support calls. Data collection took place at baseline (T1) and 12 weeks later (T2). Utilization data were tracked using a log file generated for each participant at T2. Prediction models were calculated using CART (Classification and Regression Trees), a method known mostly from the field of machine learning.

**Results:**

Acceptance, of the intervention as defined in this paper was very high on objective (89.4% have taken up the intervention) and subjective measures (91.4% reported having used the intervention and 95.3% reported they would recommend the intervention to a friend). The average number of logins corresponded to recommendations. Predictors of acceptance and predictors of utilization were similar and included, e.g., child’s externalizing symptoms, parental psychopathology, and above all additional telephone-based support by counselors.

**Conclusions:**

Through a detailed identification of acceptance and utilization, and the predictors thereof, we were able to gain a better understanding of the acceptance and utilization of web-assisted self-help for a parent management intervention in the treatment of children with ADHD and ODD. These findings can be used to recommend web-based interventions to particularly suitable families. It should be noted that some form of support is required for an intensive engagement with the content of the program.

**Trial Registration:**

The protocol of the study (German Clinical Trials Register DRKS00013456 conducted on January 3rd, 2018) was approved by the Ethics Committee of the University Hospital, Cologne.

**Supplementary Information:**

The online version contains supplementary material available at 10.1186/s13034-024-00724-0.

## Background

Parenting management training (PMT) has been shown to be effective in the treatment of children with ADHD and other externalizing disorders [1, 2]. Moreover, it has been found that behavioral PMT significantly reduces child denial behavior and noncompliance as well as parental stress [3]. Behavioral PMT aims to teach positive parenting skills in order to reduce common externalizing problems associated with ADHD symptoms in children. Generally speaking, behavioral PMT is based on cognitive behavioral strategies such as learning how to give effective commands and implementing contingency management strategies [4]. In view of the established evidence for the efficacy of (conventional) PMT, it forms part of the recommendations in current guidelines for ADHD, along with cognitive behavioral therapy and pharmacotherapy [5–7]. However, due to treatment supply gaps, which have been further compounded and magnified by the COVID-19 pandemic, there is an urgent need for evidence-based, low-cost, innovative, and easily accessible treatment approaches [8, 9].

Research has shown that innovative forms of treatment such as web- or telephone-based parental interventions can overcome some of the structural and social barriers (e.g. parental work commitments, fear of judgment) to accessing and attending parent management interventions in the treatment of child externalizing behavior problems [10, 11]. Indeed, several systematic reviews have reported that web-based PMTs are effective in reducing problem behavior, mostly with small to moderate effect sizes [12–16]. Furthermore, there is sufficient evidence that regular use and attendance is an essential prerequisite for the efficacy of any treatment, including web-based interventions [17].

In an online survey, some parents of school-aged children expressed a preference for parenting information on child mental health to be delivered via online programs rather than face-to-face training [18]. For a face-to-face PMT program, Breitenstein and coworkers found uptake rates (at least one session) of 76%, with mean attendance rates of 50% (out of 12 sessions) [19]. In later research, the same authors reported significantly higher module completion rates (85.4%) for an adapted tablet-based parent management program compared to attendance of sessions in face-to-face training [13]. In a randomized controlled trial comparing face-to-face and online behavioral parent training, DuPaul and colleagues found moderate acceptability for online PMT as rated by parents [20].

To date, however, operationalizations of the concept of acceptance of computerized interventions are heterogeneous, and measures of acceptance and utilization range from direct measures (e.g., self-report) [21] to objective measures (e.g., uptake time), with internet technologies enabling user behavior in digital health interventions to be measured and tracked. Addressing the inconsistent designation and definition of the term acceptance, Rost and colleagues concluded that “it is not an instantaneous act”, but instead a process of “accepting, experiencing, and being satisfied.” [22]. Prior to the advent of objective data tracking, empirical studies used subjective ratings by users, meaning that previous research often lacks validity, potentially resulting in over- or under-reporting of the acceptance and utilization of interventions. Nevertheless, such measures are often used to complement objective data [23]. Objective measures of utilization focus on temporal aspects (e.g., frequency, duration) and depth dimensions (e.g., amount use of specific intervention content in percentage terms) [21, 24]. However, such a one-dimensional description has been criticized given that, for example, users may be logged in for a long period of time while making little progress. Therefore, researchers have called for a multidimensional description in terms of the so-called FITT categories (i.e., frequency, intensity, time, and type of engagement), which provide concrete dimensions associated with behavior-changing aspects of an intervention. The type of engagement can in turn be distinguished into “active” use (e.g., self-monitoring, writing) or “passive” use (e.g., viewing the intervention without interacting) [25].

A recent study by Tarver et al. (2021) identified several factors that may act as barriers to the utilization of web-based PMT, such as the presence of siblings during the intervention [26]. Unfortunately, most research findings do not allow for reliable inferences regarding the direct influence of individual variables (e.g., socioeconomic status) on the utilization of web-based interventions [16]. A meta-analysis revealed that older age, higher educational attainment, and female gender of the user were relevant factors influencing engagement with digital behavior change interventions (DBCIs) in adults [21]. DuPaul and colleagues recommend focusing on possible predictors in order to provide the optimal care tailored to the needs of individual families, as their research demonstrated similar effects of face-to-face and online PMT in terms of acceptability and engagement [20]. However, in addition to sociodemographic and socioeconomic factors that may affect the utilization of web-based interventions, telephone-based support by therapists has been found to improve adherence and motivation [26]. Indeed, our own investigations showed that low-frequency telephone-based support by specialists can even improve the outcome effects of an intervention [27]. With the ultimate aim of improving the therapeutic supply situation for families with children with ADHD, the present study seeks to enhance the understanding of which families are likely to utilize web-assisted self-help.

Therefore, we investigated the acceptance and utilization of a so-called web-assisted self-help parent management training (WASH) program as part of the routine care of ADHD and other externalizing problems such as oppositional defiant disorder (ODD). A detailed and objective assessment of the parameters of utilization should provide more reliable insight into acceptance and utilization compared to previous studies that investigated self-reported usage or one-dimensional objective parameters [28]. Participants were randomized into two intervention groups (with and without additional telephone-assisted support) and compared regarding their acceptance and utilization of the online parent management training. We expected participants receiving additional telephone support to accept and use the intervention more often and intensively. In line with the literature and previous research on acceptance, we expected good overall acceptance regarding the WASH intervention. Based on previous findings and discussions, we took a multidimensional perspective to describe and analyze caregivers’ utilization of the intervention, and expected to find, for example, that socioeconomic status, single parenthood, the option of personal telephone-assisted support, and the degree of parental psychopathological problems would be associated with and predictors of acceptance and utilization of WASH for parents.

## Methods

### Study design

**Fig. 1:**
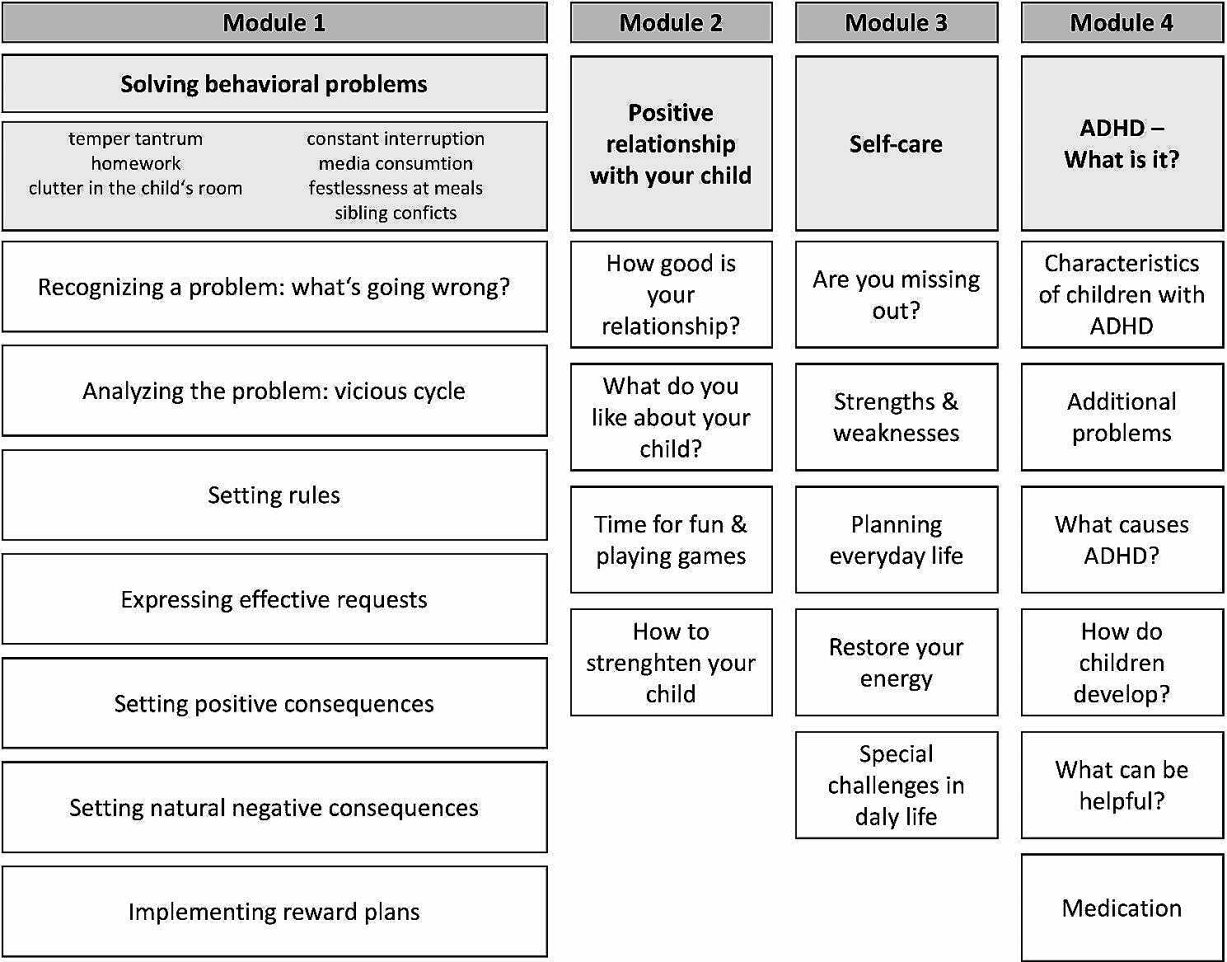
Structure of the WASH Parent Management Training (http://www.adhs.aok.de)

This analysis is based on the intervention groups of a sample recruited by pediatricians and child and adolescent psychiatrists nationwide, within a 27-month period, as part of a study to evaluate the efficacy of a web-assisted self-help program (WASH; see Fig. 1) for caregivers of children with ADHD. The study protocol of the WASH study was published prior to recruitment [29]. Families of children aged 6 to 12 years with a (suspected) diagnosis of ADHD and/or ODD (T0) could be referred by their healthcare provider (HCP). For inclusion, children’s externalizing (ADHD or ODD) symptoms had to be rated as clinically relevant at the first assessment time point (T1) by a member of the research team based on caregiver description using a semi-structured interview [30]. A diagnosis of mental retardation or autism spectrum disorder, as well as an indication for inpatient treatment, led to the exclusion of the family.

Eligible families were randomized to one of the two intervention conditions web-assisted self-help (WASH) or web-assisted self-help plus optional telephone support calls (WASH + SUPPORT), or to the control group (excluded from the present analyses). The WASH group was not actively contacted until post-assessment (T2), 12 weeks after T1. The WASH + SUPPORT group received an initial telephone call from a research team member providing information (introduction to SUPPORT, arrangement of the first appointment). SUPPORT comprised up to six 20-minute telephone calls with a trained therapist under regular supervision, in which the therapist answered any questions and provided specific advice, mainly regarding the implementation of interventions recommended by the program into daily life.

### Intervention

#### Web-assisted self-help for parents of children with ADHD (WASH)

WASH [31] is a self-help intervention for parents, which has been found to effectively reduce children’s ADHD symptoms and oppositional behavior problems [27]. It is based on the Therapy Program for Parents of Children with Hyperkinetic and Oppositional Problem Behavior, the effectiveness of which has been demonstrated in several studies, both in the form of a face-to-face intervention and as a telephone-based self-help intervention [32–39]. The intervention’s matrix design allows users to take an interest-based path through the program contents (see Fig. 1).

The program consists of four modules, each containing several components including (a) active tasks, i.e. text modules tailored to users’ answers in previous interactive tasks assessing their individual situation (e.g. quality of their relationship with the child, ability to consistently express their family rules), and (b) passive tasks, i.e. videos and audio recordings to make the content easily accessible and to present options for solutions (see Table 1). Participants were provided with password-protected access to the online intervention. Upon logging in for the first time, they received user recommendations, including an overview and guidance for working through the program in a stepwise manner within the next 12 weeks. If a participant did not log in within the first five days after gaining access, they received an email reminder. Additionally, during the first two weeks of the intervention phase, all participants received three short reminder newsletters. Users could actively choose to receive further reminders.

### Measures

Data were provided by the HCP (T0) and collected using (standardized) online questionnaires completed by the caregivers before the start of the intervention (T1) and three months after beginning the intervention (T2). Pre-intervention measures included: sociodemographic and socioeconomic status, utilization of further healthcare or child welfare services, special educational needs of the child or placement in a special school, symptoms of the child, psychopathology of the caregiver, and parenting style.

ADHD and ODD symptoms of the child were reported by the participating caregiver at T1 using the German Symptom Checklist for ADHD (SCL-ADHD) and ODD/CD (SCL-ODD) [30]. Both questionnaires consider the diagnostic criteria according to the DSM-5 and ICD-10. The SCL-ADHD assesses ADHD symptoms with eighteen items while the SCL-ODD assesses ODD symptoms with eight items. All items are rated on a four-point Likert scale ranging from 0 (not at all) to 3 (very much/particularly severe). Good internal consistency (Cronbach’s alpha) has been shown for both instruments (overall ADHD symptoms: α = 0.92; ODD symptoms: α = 0.90).

Parental mental health was assessed at T1 using the German version of the Depression Anxiety Stress Scales (DASS) [40], a 42-item self-report questionnaire comprising three 14-item scales covering symptoms of depression, anxiety, and stress. Items are rated on a four-point Likert scale ranging from 0 (did not apply to me at all) to 3 (applied to me very much, or most of the time), referring to the past week. For the present analysis, item mean scores were calculated for the three scales, demonstrating good to very good internal consistencies at T1 (Cronbach’s α = 0.95, subscales Cronbach’s α = 0.86 to 0.90).

The German Questionnaire for Positive and Negative Parenting Behavior (PNPQ) is a self-report measure assessing functional and dysfunctional parenting behavior practices [41]. Parents rate each of the 38 items (21 items on positive parenting behavior and 17 items on negative parenting behavior) on a four-point Likert scale ranging from 1 (never) to 4 (very often/most of the time). In the standardized questionnaire, positive parenting behavior is defined as that which promotes beneficial parent-child interaction, while negative parenting behavior is defined as inconsistent, impulsive, rigid parenting strategies. In the present sample, both scales showed satisfactory consistency (Cronbach’s α_positive_ = 0.86; Cronbach’s α_negative_ = 0.70).

Each participant generated log files by using the program from T1 to T2. These files were imported into SPSS for descriptive and inferential statistical analyses and into R for prediction analyses [42, 43]. Numerical aspects of utilization of the program (e.g., number of logins, duration of use, number of completed videos, number of completed tasks) for the 12-week intervention phase were extracted from the system at T2 for each user. Based on this, variables such as intervention uptake rates and uptake time, as well as frequency and process of use (in %) were aggregated. We defined *intensity of use* as the percentage of tasks/videos completed in the intervention in relation to the total number of tasks and videos within a module/ situation (see Table 1). Reliability analyses for the calculated *intensity of use* scale revealed an acceptable internal consistency (α = 0.78). *Acceptance* was defined according to the literature as follows: At T2, caregivers were asked (a) whether they had used the program and (b) whether they would recommend the program to a friend [22, 44]. In addition to these subjective measures, we recorded the following objective measures of acceptance: the *uptake rate* (i.e., at least one login) and the *return rate* (i.e., a minimum of two logins). The *uptake time* refers to the number of days between a participant receiving the login information and their first login. As the original variable measuring the *duration of use* (time in minutes) was subject to a systematic recording error (no automatic logout if the page continued to run in the background as an open tab, e.g., on a mobile device), the variable of duration of use was checked for outlier values. Therefore 9% of the training participants no longer had a valid value for the variable *duration of use* and were not included in the analyses of this dimension.

**Table 1:** Number of Tasks, Videos, and Audios in WASH per Module, Situation, and Total

Module / Situation	Content	Interactive Tasks	Videos/ Audios	Total (100%)
Module 1	Solving behavioural problems	288	46	334
Situation 1	Temper tantrum	50	8	58
Situation 2	Homework	42	7	49
Situation 3	Chaos in the child’s room	34	6	40
Situation 4	Constant interruption	43	6	49
Situation 5	Media consumption	34	6	39
Situation 6	Sibling conflicts	43	6	49
Situation 7	Restlessness at meals	42	7	49
Module 2	Positive Relationship with your child	10	8	18
Module 3	Self-Care	21	5	26
Module 4	ADHD-What is it?	7	(4)	11
Module 1–4	Total Utilization	326	59	385

### Sample

**Fig. 2:**
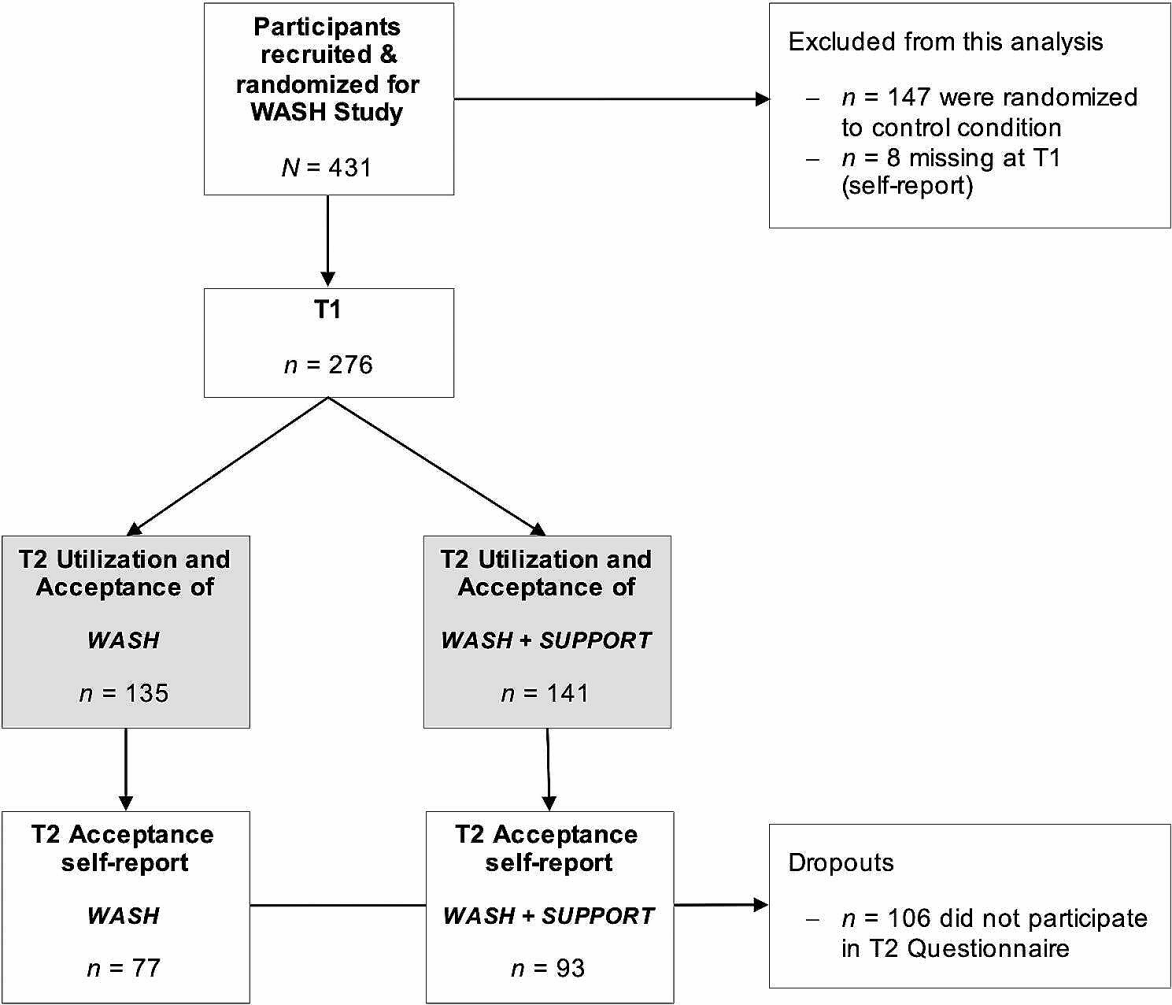
CONSORT participant flow

The following analyses were conducted in the subsample of *N* = 276 (T1) caregivers in the two intervention conditions WASH (*n* = 135) and WASH + SUPPORT (*n* = 141) who were recruited as part of the WASH study (see Fig. 2). Participants of the original WASH *sampl*e who were randomized to the control condition (*n* = 147) and cases with missing values at T1 (*n* = 8) were excluded from the following analysis. Dropout at the T2 measurement “Acceptance self-report” resulted in a reduced sample for the subjective self-report (*n* = 170) on utilization and rating of the intervention. For the present analyses, this had no impact on the following prediction of utilization, which is based on objective measurement (highlighted in grey in Fig. 2).

### Statistical methods

To gain further insight into utilization-related factors, the present article is based on exploratory, inductive data analyses using *IBM SPSS Version 28.0* [43]. For independent group comparisons of the central tendencies, methods appropriate to the scale level were used (binomial test, Mann-Whitney U test, t-test). To obtain a broad overview of multiple factors predicting the utilization of web-assisted self-help for parents, a tree analysis using the CART (Classification and Regression Trees) method was conducted [45, 46] within the statistical software *R version 4.2.1* [47]. The method of decision tree analysis has recently become popular in medical research [48]. CART visualizes complex data structures based on decision tree models, allowing for easy interpretation. It enables the splitting of various (unselected beforehand) categorical and continuous dependent variables, representing classification rules based on decision paths (“if-then”). In our analyses, the splitting procedure was continued until the following, predefined criteria were met: a minimum number of cases per final tree leaf (minbucket) = 5, the minimum number of cases allowing for a split (minsplit) = 15, the maximum number of trials to find a valid split (cross-validation) = 5. To find the best model, the largest possible tree was grown first and then pruned [48]. Hence, we focused on the predictive and non-predictive information provided by a model rather than exclusively on its performance. This allows results to be implemented and used in practice and not only in the research context. The receiver operating characteristic (ROC) analysis was conducted to evaluate the performance of the reported prediction model using the Area Under the Curve (AUC) value with regard to the 95% confidence interval (CI). The AUC indicates the accuracy, i.e. overall success rate [49]. The value defines the proportion of correctly classified records, resulting in the probability with which the prediction of acceptance or utilization can be correctly made. Generally, AUCs from 0.7 to 0.8 are rated as acceptable and AUCs from 0.8 to 0.9 as excellent [50]. In addition, to balance the method, we compared the CART analysis with hierarchical backward stepwise regression [51]. Continuous predictor variables (including number of support calls, number of sessions) were rounded to whole numbers. We conducted tree analyses with the outcome variables (1) *uptake rate* and (2) *return rate* to explore the previously described issues in research on acceptance. To predict utilization, we defined two outcome variables for conducting the tree analyses: (1) *number of logins* (n, temporal dimension as in frequency of use) and (2) *utilization* (%, depth dimension as in intensity of use). The prediction analyses were conducted using the data of the 276 caregivers who participated in the pre-assessment and were given access to the intervention (see Fig. 2).

## Results

### Descriptive characteristics of the sample

Table 2 summarizes the sample characteristics. Caregivers were mostly female (92%) and biological parents of the child (94.9%), and their mean age lay at 41.9 years (± 5.95). The majority were born in Germany (91.7%), with German as the main spoken language in the household (98.2%). 87.3% reported two children per household, which is also the numerically largest group in Germany as a whole (“one or two children”: 86.54%) [52]. In 90.9% of cases, caregivers reported that the children had a room of their own at home. Caregivers reported an employment rate of 85.5%, which is comparable to the results of the German 2019 microcensus for German mothers aged 26–61 years (97.5% employment rate). The majority of caregivers reported a medium to high level of educational attainment (university entrance-level qualification: 59.8%, with university degree: 29.0%). In general, the results indicated a high socioeconomic status.

**Table 2:** Sample characteristics of caregiver and child at T0 & T1 (*n* = 276)

			Total *n* = 276				WASH *n* = 135				WASH + SUPPORT *n* = 141				Statistics
			*M*	*SD*	Min	Max	*M*	*SD*	Min	Max	*M*	*SD*	Min	Max	*p*
**Healthcare Supply**															
	Profession (paediatrician)		163 (59.1%)				84 (62.2%)				79 (56.0%)				0.078^7^
	Supply situation (urban)		230 (83.3%)				114 (84.4%)				116 (82.3%)				0.275^7^
**Caregiver**															
	Age (years)^1^		41.93	5.95	26.61	61.54	41.70	6.03	26.61	61.54	42.15	5.90	28.61	58.78	0.530^8^
	Sex (female)		254 (92.0%)				124 (91.9%)				130 (92.2%)				0.527^9^
	Partnership/ married (yes)		244 (88.4%)				119 (88.1%)				125 (88.7%)				0.484^9^
Highest education															0.181^7^
	Low^2^		38 (13.8%)				17 (12.6%)				21 (14.9%)				
	Medium^3^		131 (47.5%)				60 (44.4%)				71 (50.4%)				
	High^4^		107 (38.8%)				58 (43.0%)				49 (34.8%)				
Currently employed (yes)			122 (85.5%)				114 (84.4%)				122 (86.5%)				0.287^9^
Parenting^5^															
	PNPQ – positive parenting		3.02	0.33	2.19	3.81	3.02	0.33	2.19	3.71	3.02	0.32	2.29	3.81	0.985^8^
	PNPQ – negative parenting		1.94	0.29	1.24	2.82	1.93	0.29	1.29	2.71	1.94	0.28	1.24	2.82	0.792^8^
Psychopathology^6^															
	DASS – total		0.61	0.40	0	2.26	0.60	0.37	0	1.83	0.62	0.43	0	2.26	0.650^8^
**Child**															
	Age (years)		9.34	1.73	6.0	12.97	9.31	1.73	6.00	12.97	9.34	1.74	6.00	12.92	0.720^8^
	Sex (male)		226 (81.9%)				109 (80.7%)				117 (83.0%)				0.286^9^
Number of siblings			1.05	0.83	0	5	1.02	0.81	0	4	1.09	0.85	0	5	0.529^8^
Externalizing symptoms															
SCL-ADHD	Total value		1.77	0.52	0.55	3.00	1.83	0.47	0.56	3.00	1.72	0.56	0.55	2.95	0.095^8^
	Inattention^6^		1.98	0.54	0.22	3.00	2.03	0.48	0.22	3.00	1.94	0.58	0.44	3.00	0.202^8^
	Hyperactivity/ impulsivity^6^		1.60	0.67	0	3.00	1.66	0.64	0	3.00	1.54	0.68	0	2.91	0.128^8^
	Skills & reflectivity		0.69	0.49	0	2.67	0.64	0.45	0	2.67	0.74	0.52	0	2.67	0.091^8^
	Impairment & burden		1.75	0.63	0.25	3.00	1.80	0.63	0.40	3.00	1.69	0.63	0.25	3.00	0.134^8^
SCL-ODD	Oppositional behavior^6^		1.50	0.69	0	3.00	1.54	0.66	0.13	3.00	1.46	0.72	0	2.75	0.361^8^
	Prosocial behavior		0.73	0.52	0	2.91	0.74	0.55	0.50	2.83	0.72	0.49	0.42	3.00	0.728^8^
	Impairment & burden		0.86	0.46	0	2.29	1.45	0.72	0	3.00	1.35	0.74	0	3.00	0.243^8^

*Note* n = sample size, *M* = mean, *SD* = standard deviation, Min = minimum, Max = maximum, *p* = probability of error, PNPQ: Positive and Negative Parenting Questionnaire, DASS: Depression, Anxiety and Stress Scales, SCL-ADHD = Caregiver Self-Checklist of Child’s Attention-deficit/ Hyperactivitiy Symptoms, SCL-ODD = Caregiver Self-Checklist of Child’s Oppositional Defiant Disorder Symptoms

^1^Reduced n due to one missing value at T0. ^2^ low = no school-leaving qualification, primary school certificate or special needs school certificate, ^3^ medium = secondary school-leaving certificate, ^4^ high = high school graduation, technical college entrance qualification, university entry qualification, technical/master school, university (of applied sciences) degree; ^5^ High key value indicates high agreement with positive/ negative parenting style. ^6^ Items rated 0 ‘not at all’, 1 ‘a little applicable’, 2 ‘largely applicable’, and 3 ‘especially applicable’. A high key value (mean item score) indicates high symptomology. ^7^ Intergroup differences were tested using Mann-Whitney U test. ^8^ Intergroup differences were tested using t-test for independent samples. ^9^ Intergroup differences were tested using binomial test

The average age of the 276 children was 9.3 years (± 1.7) and 81.9% were male. According to the information provided by the HCP at T0, 23.2% of the children had a “suspected diagnosis of AD(H)D” and 58.0% had a diagnosis of “ADHD (F90.0)”. The sex ratio in our ADHD-positive sample corresponds to the prevalence rates of ADHD in Germany, with boys being more frequently diagnosed than girls by a factor of 4.3 [53]. The majority of the children (77.2%) attended a regular school and had not received a special educational needs status. For the parent- and child-related variables, no statistical differences between the two intervention conditions were found at pre-assessment, meaning that the two conditions could be analyzed together. Previous (“never”, “only in the past”) or current (“until today”) usage of child- and parent-centered healthcare and youth welfare services was assessed with reference to the clinical guidelines (see Additional file 1). Caregiver experience with parent-centered interventions was rare (internet-based self-help interventions: 4.3%; self-help groups: 8.4%; parent management training by a specialist: 16%).

### Description of user acceptance and utilization

The data flow of objective and subjective measures of acceptance is presented in Additional file 2. Subjectively assessed rates are reported based on participants remaining in the study until T2, which seems to provide the best estimate. Objectively measures are reported based on all participants included at T1. The uptake rate (i.e., at least one login) in our sample lay at 85.9%. The uptake rate in the WASH + SUPPORT condition (89.4%) was significantly higher (binomial test: *p* = .013) than that in the non-supported WASH condition (82.2%). The return rate (i.e., at least two logins) for the whole sample was 71.4%, and the two conditions differed significantly (binomial test: *p* ≤ .001), with more returns for WASH + SUPPORT (80.1%) than for WASH only (62.2%). At post-assessment (T2, *n* = 187), caregiver self-reported (subjective measures) acceptance was high, with 91.4% (*n* = 171) reported having used the program (at least once or regularly), of which 95.3% (*n* = 163) would rather recommend the program to a friend. Again, there were significant differences between the two conditions, with WASH + SUPPORT more frequently logging into the intervention at least once (binomial test: *p* = .003) and being more likely to recommend the intervention to a friend (binomial test: *p* = .005).

Caregivers’ utilization can be described in terms of *frequency, intensity*, and *further dimensions*; intergroup differences are shown in Table 3. The majority of participants (75.9%, *n* = 167) logged in for the first time within eight days after the start of the intervention. The average *response time (uptake time)* was 8.71 days, but showed a high variance, resulting in no significant differences between the two conditions. On average, five logins (*number of logins*) were generated, with a total usage *duration* of 4.84 h. Users processed an average of 31.17% (± 26.18%) of the content across all modules, with 5% (*n* = 15) of the caregivers completing more than 75% of the content. Participants in the supported condition used the program significantly more often (t (273.79) = -3.583; *p* ≤ .001), more intensively (t (274) = -2.766; *p* = .006), and for a longer time (t (196.57) = -3.271; *p* = .001) compared to those in the pure self-help condition. Overall, between 74% and 85% of the caregivers viewed (i.e. had seen any of the content from a module) the four modules (see Additional file 3). Those who viewed any of the content from module 1 (85.9%) had also viewed modules 2 and 3. Although the psychoeducation module (module 4) was viewed by slightly fewer caregivers (74%), it was processed the most (53% on average). We found statistically significant correlations between the utilization variables (global as well as detailed) and other described usage parameters, lying in the expected positive direction (see Additional file 4). The strongest (positive) correlation was found between *intensity* (%) and *frequency of use* (*r* = .73, *p* ≤ .001). However, other correlations were weak to moderate. The corrected time variable “duration” (variable corrected for outliers as described above), as with the correlations for all other variables, showed moderate significant correlations with the other usage parameters (r_*intensity*_=0.39, *p* ≤ .001; r_*frequency*_=0.43, *p* ≤ .001) and can thus be regarded as a reliable measure and description of temporal utilization. Moreover, significant correlations emerged between active (individual rating or text) and passive (video or audio) utilization for all modules (see Additional file 5) and in total (*r* = .72, *p* ≤ .001). However, mean comparisons indicate more usage (intensity) of passive than active steps.

**Table 3:** Utilization parameters and intergroup differences

Parameters of Utilization	Total					WASH					WASH + SUPPORT					Statistics		
	*n*	*M*	*SD*	Min	Max	*n*	*M*	*SD*	Min	Max	*n*	*M*	*SD*	Min	Max	*t*	*df*	*p* ^*1*^
Frequency																		
Number of logins (n)	276	5.00	4.38	0	18	135	4.05	4.25	0	17	141	5.90	4.32	0	18	-3.583	273.79	< 0.001
Intensity																		
Process (%)	276	31.88	26.08	0	96.70	135	26.97	25.58	0	96.70	141	36.58	25.78	0	88.07	-2.766	274	0.006
Further Parameters																		
Uptake time (days)	237	8.71	14.63	0	82	111	8.62	14.67	0	82	126	8.79	14.65	0	79	− 0.086	235	0.930
Duration (h)	254	4.84	8.20	0	53.87	126	3.18	5.42	0	31.95	128	6.48	9.98	0	53.87	-3.271	196.57	0.001
Support calls (n)	-	-	-	-	-	-	-	-	-	-	143	4.62	2.09	0	6	-	-	-

*Note*^1^For group differences, a t-test for independent groups was calculated

### Prediction analyses

For the prediction analyses, significant main decision paths are reported. A full overview of all decision paths (for all decision trees conducted in this analysis) can be retrieved from Additional file 6.

### Acceptance

**Fig. 3:**
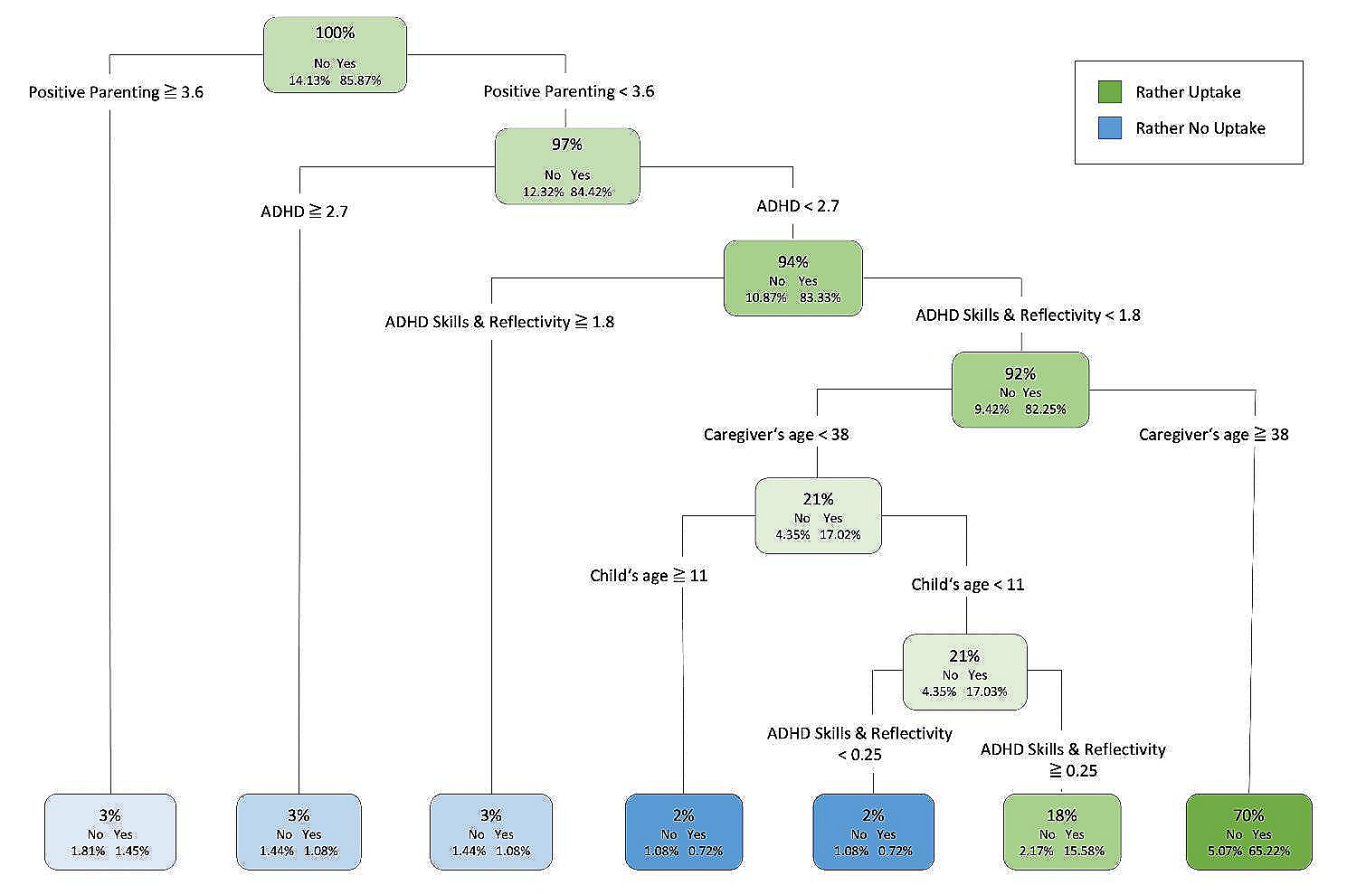
Decision tree predicting initial uptake of the WASH intervention. *Notes.* For each node, the first line shows the percentage of our sample (*n* = 276) that ends up in the leaf, followed by the information whether the intervention was initially taken up (yes) or not (no) and the third line indicating the respective rate in relation to the sample. The color of leaf indicates that the majority of cases in this leaf (paths) has taken up (green) or has not taken up (blue); ADHD = ADHD Overall Symptomatology

To predict initial parental *acceptance* of the intervention, we conducted a CART analysis on the variable *uptake*, with an acceptable AUC of 0.74 (CI: 0.65–0.83) for the biggest tree grown (see Fig. 3). The best primary predictor of uptake was positive parenting skills measured at T1. Overall, it was observed that initial uptake was more likely for 65.2% (*n* = 180) of the sample who reported a positive parenting (PNPQ) score below 3.6, an ADHD symptom score below 2.7 (range 0–3), a score below 1.8 on the FBB-ADHS subscale assessing competent behavior of endurance, attention, and reflectivity (‘ADHD Skills & Reflectivity’, range 0–3), and with a caregiver age ≥ 38 years. However, approximately 5% of these cases still did not take up the intervention. Relatively speaking, initial uptake was also more likely for a further 18% of the sample with a caregiver age below 38 years, a child age below 11 years, and a score of 0.25 or higher on the SCL-ADHD subscale assessing competent behavior of endurance, attention, and reflectivity.

**Fig. 4:**
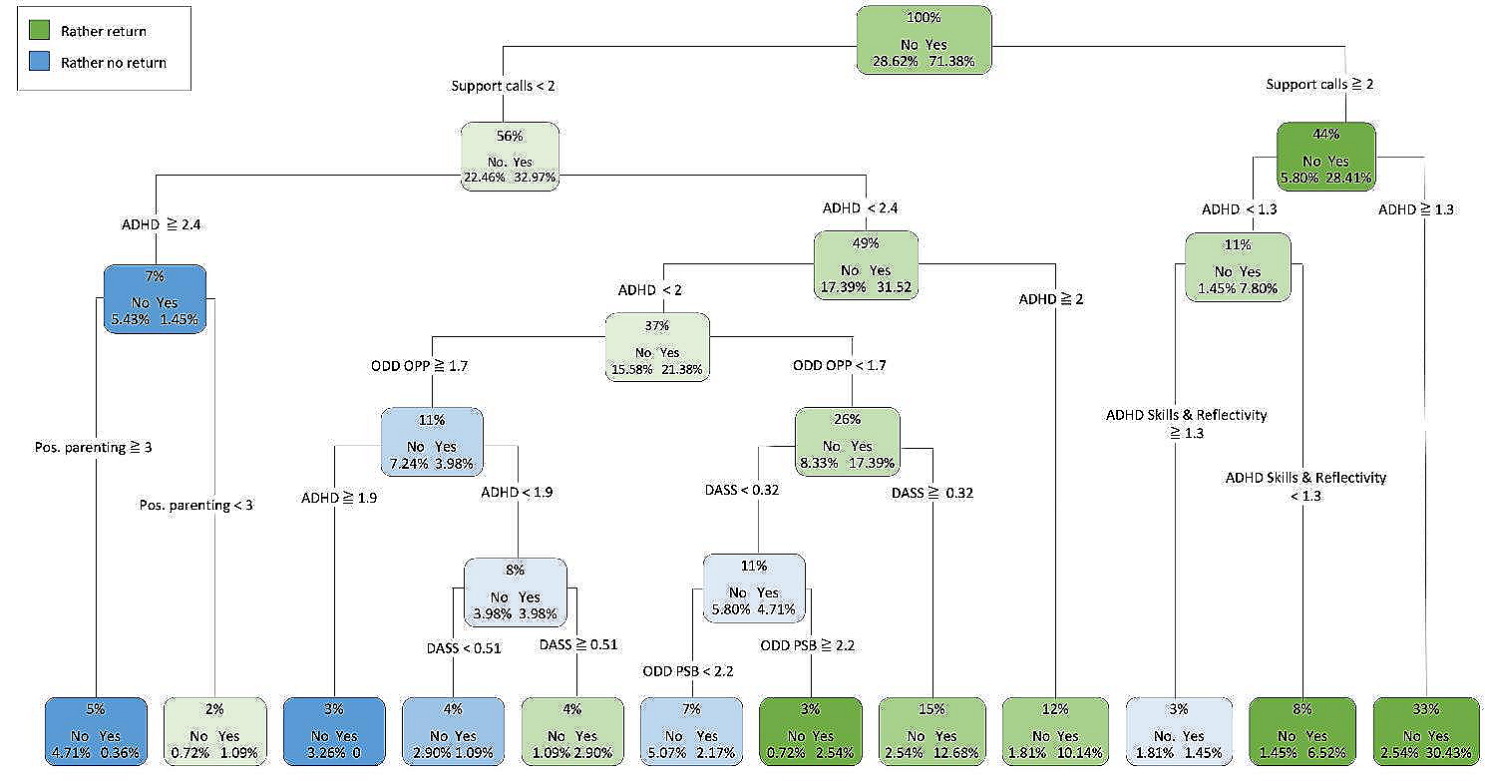
Decision tree predicting a return to the intervention after initial login. *Notes.* For each node, the first line shows the percentage of our sample (*n* = 276) that ends up in the leaf, followed by the information whether caregivers returned to the intervention after initial login (yes) or not (no) and the third line indicating that the respective rate is in relation to the sample. The color of leaf indicates the majority of cases in this leaf (paths) returned (green) or did not return (blue); ADHD = ADHD Overall Symptomatology, ODD OPP = Oppositional Defiant Disorder, Oppositional Subscale; ODD PSB = Oppositional Defiant Disorder, Prosocial Behavior; DASS = Parental Psychopathology (Depression, Anxiety, Stress), Pos. Parenting = Positive Scale

The starting point of CART for the dependent variable *return rate*, as another index of acceptance, is the number of support calls, indicating that this primary split is the best predictor of returning to the intervention after the initial uptake (see Fig. 4). For 33% of the sample, the results indicate that with two or more support calls and a caregiver-reported ADHD symptom score above 1.3, 30.4% returned to the intervention and only 2.5% did not return. A stronger tendency towards *not returning* to the intervention was predicted for 4.7% of our sample with less than two support calls, a very high caregiver-reported ADHD score (over 2.4), and very positive parenting (≥ 3). In this decision path, only *n* = 1 caregiver returned to the intervention. Further factors such as the child’s oppositional symptoms and prosocial behavior as well as parental psychopathology (depression, stress, anxiety) added to the prediction of parental return to WASH. However, other factors, such as supply situation, type of intervention (WASH or WASH + SUPPORT), and sociodemographic and socioeconomic variables of the caregiver or child were not significantly related to whether caregivers returned to the intervention after an initial login. The result, with an excellent AUC of 0.84 (CI: 0.78–0.89), correctly predicts 84% of the records regarding whether or not users returned.

### Prediction of frequency and intensity of use

**Fig. 5:**
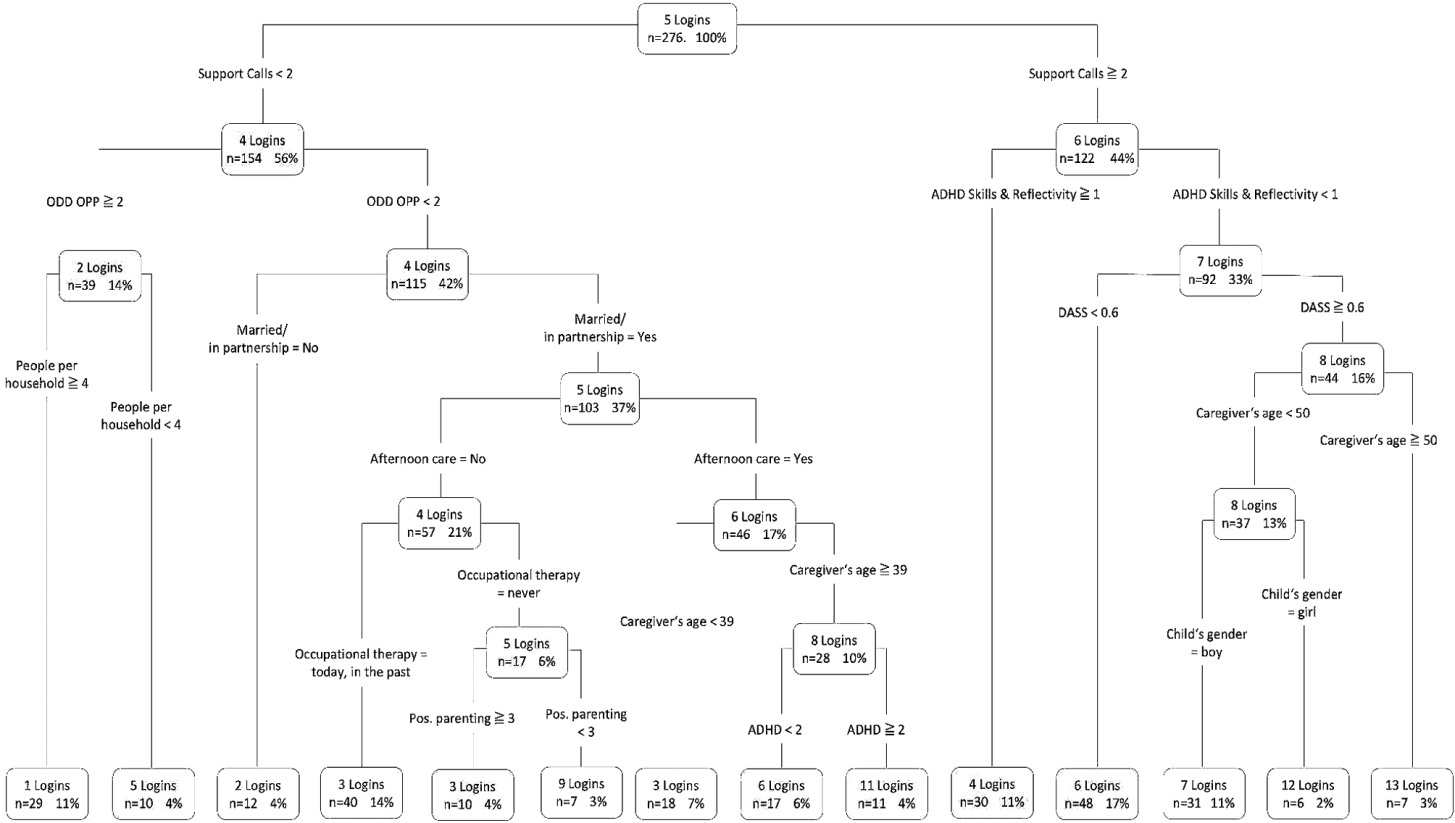
Decision tree predicting the frequency of use (number of logins, n). *Notes.* For each node, the first line shows the *mean numbers of logins*. In the second line, the number of cases and percentage of the complete sample (*n* = 276) that ends up in the branch is displayed. ADHD = ADHD Overall Symptomatology; ODD OPP = Oppositional Defiant Disorder, Oppositional Subscale; DASS = Parental Psychopathology (Depression, Anxiety, Stress), Pos. Parenting = PNPQ, Positive Scale

The *frequency of use* (i.e., number of logins; see Fig. 5) was predicted with an excellent accuracy of AUC = 0.80 (CI: 0.75–0.85). The branching nodes show the mean number of logins (first line in each node) and the absolute and relative number of caregivers ending up in the branch (second line in each node). The mean number of logins across all parents (*n* = 276) was 5. The decision rules can be divided into decision paths that lead to very frequent usage (more than 10 logins) and very little usage (maximum of two logins). Besides the number of support calls, the symptoms of the child (ADHD and ODD symptoms), and parental psychopathology, it emerged that sociodemographic variables of the child (gender) and caregiver (age), as well as socioeconomic variables (e.g., afternoon/evening childcare, marriage status) and parenting skills (positive parenting) were meaningful predictors of caregivers’ number of logins to the intervention. Above all, the starting point of the tree is the number of support calls, which affected parental utilization in terms of frequency. However, even when less than two support calls were realized, some further factors positively affected the frequency of use: An above-average number of logins (e.g. eleven logins) occurred if caregivers reported a score below 2.0 for oppositional defiant disorder of the child (ODD OPP), being married or in a partnership, availability of afternoon care, caregiver’s age ≥ 39 years, and a score over 2.0 for ADHD symptoms. Other factors, such as the referring HCP (psychiatrist or pediatrician), as well as the type of intervention (WASH or WASH + SUPPORT), educational level, employment situation, and special educational needs, were not significantly related to the frequency of use.

**Fig. 6:**
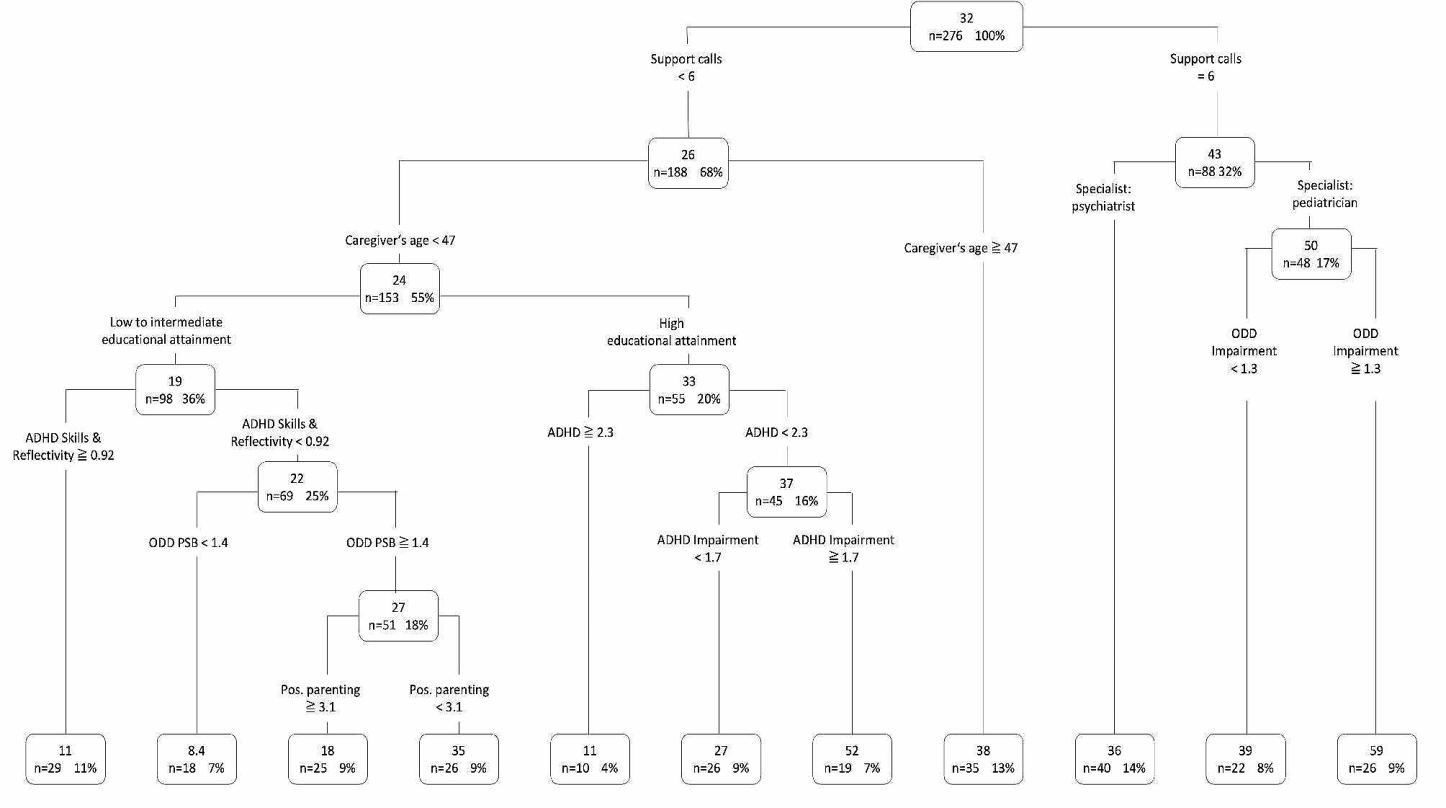
Decision tree predicting the intensity of use (completed tasks/ videos, %). *Notes.* For each node, the first line shows the *mean intensity of utilization (%)*. In the second line, the number of cases and percentage of the complete sample (*n* = 276) that ends up in the branch, is displayed. ADHD = ADHD Overall Symptomatology; ODD PSB = Oppositional Defiant Disorder, Prosocial Behavior; Pos. Parenting = PNPQ, Positive Scale

The decision tree for the *intensity of use (percentage of completed tasks/videos)* was also built with an excellent AUC of 0.80 (CI: 0.75–0.85). Figure 6 displays the mean percentage of completed tasks/videos (first number in each node) and the absolute and relative number of caregivers who achieved this completion rate (second line in each node). Across all participating caregivers, the mean percentage of completed tasks/videos was 32%. The best predictor was the number of support calls received. The decision tree splits the sample into two branches: The caregivers who used all six support calls (the maximum we offered) completed more tasks/videos than those who used fewer support calls. However, some conditions resulted in a higher rate of completed tasks/videos even for caregivers using fewer than six support calls (e.g., caregiver age ≥ 47 years). In addition to the referral context (referred by pediatrician or psychiatrist), sociodemographic (age) and socioeconomic (education) variables of the caregiver, symptoms, and impairment of the child (ADHD and ODD), and parenting skills (positive parenting) added to the prediction of caregivers’ completion rate of tasks/videos. However, the type of intervention (WASH or WASH + SUPPORT), employment situation, and other socioeconomic factors (e.g., single-parent status, number of people per household, afternoon childcare) or special educational needs, were not significantly related to the intensity of use.

For all analyses, the comparison using alternative backward stepwise regression analyses resulted in a better performance of the CART algorithm (AUC values).

## Discussion

To the best of our knowledge, the present study is the first to systematically investigate and describe factors of parental acceptance and utilization in the context of web-assisted self-help interventions for parents of children with ADHD. Our results demonstrate that (1) acceptance of WASH is high, both according to objectively collected data and self-report, and (2) the additional support calls as well as child- and caregiver-related psychopathology and positive parenting are the main predictors of acceptance and utilization. Unfortunately, like previous research, we were not able to include the underserviced target group of caregivers in less served regions, with less educational background and families with less access to evidence based ADHD treatment. Our sample has a rather high socioeconomic status, which should be considered in the following discussion and especially regarding the generalizability of the results.

Our data reveal that WASH is well accepted for both active intervention groups (with and without telephone support), as documented both by objective measures (uptake rate and return rate: from 70 to 85%) and subjective measures (self-report: 90–95%). The uptake rate was comparable to the rate of 76.4% (i.e., attending at least one session) reported in the Chicago Parent Program, a face-to-face parent training program for families from low-income urban communities [19]. Our results fit well with other web-based treatments, such as the ezParent program to prevent child behavior problems in preschoolers, in which only a third of the participants who signed up, from a low-income population, failed to attend any sessions [13]. In fact, in the present study, only 15% of participants did not log in to the WASH program at all, indicating that acceptance in our sample was higher than that in the ezParent program. These good acceptance rates may be attributable to the advantages of online interventions: WASH is available 24/7 and can be easily accessed through a smartphone, tablet, or laptop and from anywhere with internet access. Previous analyses have likewise indicated a preference for online treatment for caregivers of children with anxiety and depression [18]. Flexibility in parent training, as a key element of acceptance, was also reported by Tarver and colleagues in qualitative analyses of an ADHD self-help program [54]. It has to be taken into account, that the ezParent program is an intervention addressing families of cultural diversity and with particular needs. Therefore, comparisons of the results should be interpreted with caution. Regarding the initial login (uptake), using CART, we were able to correctly predict acceptance for about 74% of cases, which is considered acceptable [50]. The prediction of the return rate showed an even better performance, with 84% correct predictions. It has been expected that online interventions such as WASH may primarily be accessed by those in less well-served supply situations such as rural areas. However, our results did not support this assumption: For both initial uptake and return, ADHD/ODD symptoms of the child, caregiver psychopathology (depression, anxiety, and stress), and parenting style were important predictors. The most important predictor of return was the number of support calls, with a higher number of calls increasing the probability of return, in line with earlier research findings [22]. For a small group of caregivers, a very high level of self-reported positive parenting predicted non-acceptance in terms of no initial uptake. This predictor was also found in the decision paths for the return rates, indicating that caregivers with high levels of positive parenting (combined regarding return rate with less than two support calls and higher levels of ADHD) might not expect the program to be helpful for them. Moreover, even when they do accept the intervention, they tend to use it less in terms of frequency and intensity (cf. Figs. 5 and 6), supporting the hypothesis that the intervention is not very promising in this population.

Overall, research on acceptance has yielded divergent findings to date, which might be explained by the very different operationalizations of the term acceptance itself. Indeed, many research groups interpret it more in the sense of satisfaction with an intervention, rendering it difficult to compare the research findings on (online) PMT acceptance. For instance, a recent study examining behavioral parent training (BPT) with additional child-therapist support reported high acceptance based on caregivers’ satisfaction (e.g. “The length of the treatment program met my expectations and the needs of my family”), which caregivers self-rated with mean scores between 3.8 and 4.9 on a five-point Likert scale [55]. Generally speaking, there does not yet appear to be any agreed-upon best practice when it comes to evaluating patient/ user acceptance in the context of internet-/mobile-based health interventions [56]. Future research should assess acceptance based on theoretical models such as the well-established *Unified Theory of Acceptance and Use of Technology* [57], which is often reported as both theory-based and as empirically proven for the evaluation of acceptance (in technology) and focuses on aspects such as attitudes towards the technology, self-efficacy (health- and technology-related), and perceived barriers (such as security barriers).

Regarding the utilization of WASH, an average frequency of five logins (out of six recommended sessions) was found, which can be described as an 83.3% attendance rate. A study analyzing a comparable training program for parents of children with ADHD, provided either face-to-face or online, reported a mean of 80% session attendance/ completion, which the authors described as “high”, and a t-test indicated no significant group differences between the online and the face-to-face condition [20]. On the other hand, the utilization in terms of completed tasks/videos in the present study is rather low (average of 31.7%). However, participants of WASH were free to choose how they engaged with the program (in terms of logins and which tasks/videos they completed) and a 100% completion rate was not expected due to the modular architecture of the website, allowing parents to only use the tasks/videos they perceived as relevant to them. A systematic review on the use of technology and digital delivery methods of parent management training reported a content completion rate ranging from 41.7 to 99.2% [58].

The decision tree models for utilization of WASH were able to correctly predict for 80% how often (number of logins) and how intensively (number of completed tasks/videos) the intervention was used. In these analyses, telephone-based support was the most important predictive variable for both utilization parameters. The influence of the number of support calls is more relevant than simply the offer of support (in one intervention group), as this variable had the same likelihood of becoming a predictor in the CART models but was not included. These findings are in line with previous research in smaller samples [26]. With comparably little support (at least two support calls), the frequency of use increased significantly, whereas more intensive support (six support calls) led to an improvement in intensity of use. However, when discussing the relevance of this variable, it is necessary to consider further variables found in the decision paths. As the efficacy of a treatment depends on its actual use, and the mere attendance of an intervention does not ensure positive intervention outcomes [17, 59], additional therapeutic support is necessary to enhance utilization. Moreover, the results provide initial indications that a differentiated consideration of different utilization parameters certainly appears to be useful. Some variables are related to both parameters, as a higher age of the user of an online intervention was found to be associated with more frequent and more intensive utilization, thus confirming previous research findings of a meta-analysis on different interventions [21]. This seems surprising, and contradicts previous findings on caregivers’ engagement in an internet-based health intervention (addressing a child’s asthma), in which younger caregivers were found to log in more often [60].

Some variables, however, were exclusively relevant for either frequency or intensity of use: The presence of siblings in the same household is one factor that may prevent caregivers from using web-based interventions [54]. Using CART, we were able to show that the number of persons per household did affect caregivers’ frequency of use (but not the intensity). It is important to note that expected factors such as being in a partnership/ married, and in line with previous research [26], availability of afternoon childcare, were predictively associated with more frequent logins, indicating that the intervention may have been more easily accessible for these families. Exclusively (for the group of caregivers aged below 47 years), educational attainment was found to predict the intensity of use, insofar as caregivers with an especially high educational status used the intervention more.

### Limitations

In terms of the findings mentioned above, it is important to note that the socioeconomic status of our sample was rather high, and the distribution of social parameters was skewed. In line with a previous study examining the role of parental engagement in a parenting program aimed at reducing risk factors of child depression and anxiety [61], we reached a mainly female, middle-aged, educationally highly qualified sample. This will primarily be an effect of the referral context (clinical utilization sample assigned by practicing psychiatrists or pediatricians), with the attractiveness of WASH for a selective sample only being a secondary factor in this regard.

The WASH study was able to make an important contribution to the empirical evaluation of factors of acceptability and utilization of web-assisted self-help. A strength of this analysis is the automatic data tracking, which enabled objective information on the acceptance and usage of the website. However, the tracking of utilization did not allow for any statements about the order of usage. Future studies should thus endeavor to record the utilization of modules in chronological order, in order not only to explain the general effectiveness but also to specify which contents of cognitive behavioral web-assisted self-help in ADHD parent management training are effective. Furthermore, we cannot guarantee that users actually engaged with the content rather than merely “clicking through” the intervention, as we did not conduct knowledge quizzes to prove caregivers’ engagement.

Using the method of CART analysis, we were certainly able to follow an inductive approach and consider diverse predictive variables for the utilization of WASH and how they are related to each other, instead of investigating pre-selected predictors. A visual comparison of the two decision trees (frequency and intensity of use) reveals differences that might hint at multiple ideas for optimizing the intervention, but should not be over-interpreted due to the limited power of the sample.

In conclusion, it seems advisable to include different parameters, as there are clear differences between objective and subjective measurements, indicating that an under- or overestimation of acceptance (and potentially utilization as well) occurs when approaches exclusively use subjective measures. Moreover, we were not able to include information on why caregivers did not use the intervention due to missing data; causes of non-acceptance and factors of drop-out should be considered in future research. Further research is needed to assess the efficacy of specific content and individual modules of the intervention and to further illuminate the important role of the support calls, especially regarding the intensity or frequency of support. The decision tree findings provide a useful contribution to the discussion of whether, and for whom, web-assisted self-help interventions can be helpful. Our results clearly underline the need for targeted recommendations oriented to specific criteria (e.g., with the help of decision tree findings), and highlight that support from a specialist who provides individual guidance throughout the intervention (main predictor) is especially important when attending web-based training, and renders the actual utilization of the intervention more likely.

### Electronic supplementary material

Below is the link to the electronic supplementary material.

**Supplementary Material 1**: Child- and Parent-Related Healthcare Services Usage

**Supplementary Material 2**: Measures of acceptance

**Supplementary Material 3**: Content, Views and Intensity of Use (%) per Module

**Supplementary Material 4**: Correlation of Utilization Dimensions

**Supplementary Material 5**: Active and Passive Utilization

**Supplementary Material 6**: Decision Paths for CART Decision Trees

## Data Availability

The datasets used and analyzed during the current study are available from the corresponding author upon reasonable request.

## Abbreviations

WASH: web-assisted self-help
SUPPORT: telephone-based support calls
RCT: randomized controlled trials
CART: Classification and Regression Trees
ADHD: Attention Deficit Hyperactivity Disorder (Symptomatology)
ODD: Oppositional Defiant Disorder
ODD OPP: Oppositional Defiant Disorder, Oppositional Scale (SCL-ODD)
ODD PSB: Oppositional Defiant Disorder, Prosocial Behavior Scale (SCL-PBS)
HCP: Healthcare Provider
PMT: Parent Management Training
FITT: Frequency, Intensity, Time, and Type of Engagement
DBCI: Digital Behavior Change Intervention
SPSS: Statistical Package for the Social Sciences
ROC: receiver operating characteristic
AUC: Area Under the Curve
CI: confidence interval
PNPQ: Positive and Negative Parenting Questionnaire
DASS: Depression, Anxiety, and Stress Scales
SCL-ADHD: Parent-rated German Symptom Checklists for ADHD
SCL-ODD: Parent-rated German Symptom Checklists for ODD
ICD-10: tenth edition of the International Statistical Classification of Diseases and Related Health Problems

## References

[1] Dekkers TJ, Hornstra R, van der Oord S, Luman M, Hoekstra PJ, Groenman AP, van den Hoofdakker BJ (2022). Meta-analysis: which components of parent training work for children with Attention-Deficit/Hyperactivity disorder?. J Am Acad Child Adolesc Psychiatry.

[2] Fabiano GA, Schatz NK, Aloe AM, Chacko A, Chronis-Tuscano A (2015). A systematic review of Meta-analyses of Psychosocial Treatment for Attention-Deficit/Hyperactivity disorder. Clin Child Fam Psychol Rev.

[3] Ciesielski HA, Loren REA, Tamm L (2020). Behavioral parent training for ADHD Reduces Situational Severity of Child Noncompliance and related parental stress. J Atten Disord.

[4] Chronis AM, Chacko A, Fabiano GA, Wymbs BT, Pelham WE (2004). Enhancements to the behavioral parent training paradigm for families of children with ADHD: review and future directions. Clin Child Fam Psychol Rev.

[5] Taylor E, Döpfner M, Sergeant J, Asherson P, Banaschewski T, Buitelaar J, Coghill D, Danckaerts M, Rothenberger A, Sonuga-Barke E (2004). European clinical guidelines for hyperkinetic disorder -- first upgrade. Eur Child Adolesc Psychiatry.

[6] Dalrymple RA, Maxwell LM, Russell S, Duthie J (2020). NICE guideline review: attention deficit hyperactivity disorder: diagnosis and management (NG87). Arch Dis Childhood-E.

[7] National Institute for Health and Care Excellence. : National Institute for Health and Care Excellence: Guidelines. In: Attention deficit hyperactivity disorder: diagnosis and management edn. London: National Institute for Health and Care Excellence (NICE) Copyright © NICE 2018.; 2019. 29634174

[8] Cortese S, Asherson P, Sonuga-Barke E, Banaschewski T, Brandeis D, Buitelaar J, Coghill D, Daley D, Danckaerts M, Dittmann RW (2020). ADHD management during the COVID-19 pandemic: guidance from the European ADHD Guidelines Group. Lancet Child Adolesc Health.

[9] Ravens-Sieberer U, Kaman A, Otto C, Adedeji A, Devine J, Erhart M, Napp AK, Becker M, Blanck-Stellmacher U, Löffler C (2020). Mental Health and Quality of Life in Children and adolescents during the COVID-19 pandemic-results of the Copsy Study. Dtsch Arztebl Int.

[10] Dose C, Hautmann C, Buerger M, Schuermann S, Woitecki K, Doepfner M (2017). Telephone-assisted self-help for parents of children with attention-deficit/hyperactivity disorder who have residual functional impairment despite methylphenidate treatment: a randomized controlled trial. J Child Psychol Psychiatry.

[11] Friars P, Mellor D (2009). Drop-out from parenting training programmes: a retrospective study. J Child Adolesc Ment Health.

[12] Baumel A, Pawar A, Kane JM, Correll CU (2016). Digital Parent Training for Children with disruptive behaviors: systematic review and Meta-analysis of Randomized trials. J Child Adolesc Psychopharmacol.

[13] Breitenstein SM, Fogg L, Ocampo EV, Acosta DI, Gross D (2016). Parent use and efficacy of a Self-Administered, Tablet-based parent training intervention: a Randomized Controlled Trial. JMIR Mhealth Uhealth.

[14] Daley D, Van Der Oord S, Ferrin M, Cortese S, Danckaerts M, Doepfner M, Van den Hoofdakker BJ, Coghill D, Thompson M, Asherson P (2018). Practitioner review: current best practice in the use of parent training and other behavioural interventions in the treatment of children and adolescents with attention deficit hyperactivity disorder. J Child Psychol Psychiatry.

[15] Sourander A, McGrath PJ, Ristkari T, Cunningham C, Huttunen J, Lingley-Pottie P, Hinkka-Yli-Salomäki S, Kinnunen M, Vuorio J, Sinokki A (2016). Internet-assisted parent training intervention for disruptive behavior in 4-Year-old children: a Randomized Clinical Trial. JAMA Psychiatry.

[16] Baumel A, Pawar A, Mathur N, Kane JM, Correll CU (2017). Technology-assisted parent training programs for children and adolescents with disruptive behaviors: a systematic review. J Clin Psychiatry.

[17] Chiu TM, Eysenbach G (2010). Stages of use: consideration, initiation, utilization, and outcomes of an internet-mediated intervention. BMC Med Inf Decis Mak.

[18] Sim WHJ, Jorm AE, Yap AF. M.: The impact and reach of web-based parenting guidelines to prevent childhood depression and anxiety: findings from online user surveys. Mental Health & Prevention; 2017.

[19] Breitenstein SM, Gross D, Fogg L, Ridge A, Garvey C, Julion W, Tucker S (2012). The Chicago parent program: comparing 1-year outcomes for African American and latino parents of young children. Res Nurs Health.

[20] DuPaul GJ, Kern L, Belk G, Custer B, Daffner M, Hatfield A, Peek D (2018). Face-to-face Versus online behavioral parent training for Young children at risk for ADHD: Treatment Engagement and outcomes. J Clin Child Adolesc Psychol.

[21] Perski O, Blandford A, West R, Michie S (2017). Conceptualising engagement with digital behaviour change interventions: a systematic review using principles from critical interpretive synthesis. Transl Behav Med.

[22] Rost T, Stein J, Löbner M, Kersting A, Luck-Sikorski C, Riedel-Heller SG (2017). User Acceptance of Computerized cognitive behavioral therapy for Depression: systematic review. J Med Internet Res.

[23] Boase J, Ling R (2013). Measuring Mobile phone use: self-report versus Log Data. J Computer-Mediated Communication.

[24] Couper MP, Alexander GL, Zhang N, Little RJA, Maddy N, Nowak MA, McClure JB, Calvi JJ, Rolnick SJ, Stopponi MA (2010). Engagement and Retention: measuring breadth and depth of participant use of an online intervention. J Med Internet Res.

[25] Short CE, DeSmet A, Woods C, Williams SL, Maher C, Middelweerd A, Muller AM, Wark PA, Vandelanotte C, Poppe L (2018). Measuring Engagement in eHealth and mHealth Behavior Change interventions: viewpoint of methodologies. J Med Internet Res.

[26] Tarver J, Daley D, Sayal K. A self-help version of the New Forest Parenting Programme for parents of children with attention deficit hyperactivity disorder: a qualitative study of parent views and acceptability. Child Adolesc Ment Health 2021. 10.1111/camh.12476 34041842

[27] Döpfner M, Plück J, Rosenberger K, Klemp M-T, Mühlenmeister J, Wähnke L, Hellmich M, Dose C. Efficacy of Guided and Unguided Web-Assisted Self-Help for Parents of Children with Externalizing Behavior Disorder: A Three-Arm Randomized Controlled Trial. submitted. 10.1111/jcpp.14153 PMC12350813 40062662

[28] Sanders MR, Dittman CK, Farruggia SP, Keown LJ (2014). A comparison of online versus workbook delivery of a self-help positive parenting program. J Prim Prev.

[29] Döpfner M, Wähnke L, Klemp M-T, Mühlenmeister J, Schürmann S, Hellmich M, Plück J (2020). Efficacy of web-assisted self-help for parents of children with ADHD (WASH) - a three-arm randomized trial under field/routine care conditions in Germany. BMC Psychiatry.

[30] Döpfner M (2017). Diagnostik-System für psychische Störungen Nach ICD-10 und DSM-5 für Kinder- Und Jugendliche - III.

[31] ADHS-Elterntrainer [Available. from https://adhs.aok.de/ [cited 2019 7 Nov].].

[32] Döpfner M, Breuer D, Schürmann S, Metternich TW, Rademacher C, Lehmkuhl G (2004). Effectiveness of an adaptive multimodal treatment in children with attention-deficit hyperactivity disorder -- global outcome. Eur Child Adolesc Psychiatry.

[33] Döpfner M (2019). Therapieprogramm für Kinder Mit hyperkinetischem und oppositionellem Problemverhalten THOP. 69 469.

[34] Döpfner M, Liebermann-Jordanidis H, Kinnen C, Hallberg N, Mokros L, Benien N, Mütsch A, Schürmann S, Wolff Metternich-Kaizman T, Hautmann C et al. Long-term effectiveness of guided self-help for parents of children with ADHD in Routine Care-An Observational Study. J Atten Disord 2018:1087054718810797. 10.1177/1087054718810797 30449268

[35] Katzmann J, Hautmann C, Greimel L, Imort S, Pinior J, Scholz K, Döpfner M (2017). Behavioral and nondirective guided self-help for parents of children with externalizing behavior: mediating mechanisms in a Head-To-Head comparison. J Abnorm Child Psychol.

[36] Hautmann C, Dose C, Duda-Kirchhof K, Greimel L, Hellmich M, Imort S, Katzmann J, Pinior J, Scholz K, Schürmann S (2018). Behavioral Versus Nonbehavioral guided self-help for parents of children with externalizing disorders in a Randomized Controlled Trial. Behav Ther.

[37] Mokros L, Benien N, Mütsch A, Kinnen C, Schürmann S, Metternich-Kaizman TW, Breuer D, Hautmann C, Ravens-Sieberer U, Klasen F (2015). [Guided self-help interventions for parents of children with ADHD–concept, referral and effectiveness in a nationwide trial. An observational study]. Z Kinder Jugendpsychiatr Psychother.

[38] Kierfeld F, Döpfner M (2006). Bibliotherapie als Behandlungs- möglichkeit Bei Kindern Mit Externalen Verhaltensstörungen. Zeitschrift für Kinder- und Jugendpsychiatrie Und Psychotherapie.

[39] Kierfeld F, Ise E, Hanisch C, Görtz-Dorten A, Döpfner M (2013). Effectiveness of telephone-assisted parent-administered behavioural family intervention for preschool children with externalizing problem behaviour: a randomized controlled trial. Eur Child Adolesc Psychiatry.

[40] Lovibond PF, Lovibond SH (1995). The structure of negative emotional states: comparison of the Depression anxiety stress scales (DASS) with the Beck Depression and anxiety inventories. Behav Res Ther.

[41] Imort S, Hautmann C, Greimel L. Der Fragebogen zum positiven und negativen Erziehungsverhalten (FPNE): Eine psychometrische Zwischenanalyse.: Poster zum 32 Symposium der Fachgruppe Klinische Psychologie und Psychotherapie der Deutschen Gesellschaft für Psychologie, Braunschweig. In.; 2014.

[42] R. : A language and environment for statistical computing. R Foundation for Statistical Computing [https://www.R-project.org/].

[43] Corp I. IBM SPSS statistics for Windows, Version 28.0. Armonk. In. NY: IBM Corp; Released 2021.

[44] Lara MA, Patiño P, Tiburcio M, Navarrete L (2022). Satisfaction and acceptability ratings of a web-based self-help intervention for Depression: retrospective cross-sectional study from a resource-limited country. JMIR Form Res.

[45] McArdle JJ, Ritschard G (2014). Contemporary issues in exploratory data mining in the behavioral sciences.

[46] Segura-Morales M, Loza-Aguirre E. Using Decision Trees for Predicting Academic Performance Based on Socio-Economic Factors; 2017.

[47] Li B, Friedman J, Olshen R, Stone C (1984). Classification and regression trees (CART). Biometrics.

[48] Song YY, Lu Y (2015). Decision tree methods: applications for classification and prediction. Shanghai Arch Psychiatry.

[49] Dietrich D, Heller B, Yang B. Data science & big data analytics: discovering, analyzing, visualizing and presenting data. Wiley; 2015.

[50] Hosmer DW Jr, Lemeshow S, Sturdivant RX. Applied logistic regression. Volume 398. Wiley; 2013.

[51] Zou KH, O’Malley AJ, Mauri L (2007). Receiver-operating characteristic analysis for evaluating diagnostic tests and predictive models. Circulation.

[52] Destatis SB. Statistischer Bericht: Mikrozensus - Haushalte und Familien In: *2021* Edited by (Destatis) SB. Wiesbaden; 2022.

[53] Schlack R, Hölling H, Kurth BM, Huss M (2007). Die Prävalenz Der Aufmerksamkeitsdefizit-/Hyperaktivitätsstörung (ADHS) bei Kindern Und Jugendlichen in Deutschland. Bundesgesundheitsblatt - Gesundheitsforschung - Gesundheitsschutz.

[54] Tarver J, Daley D, Sayal K (2022). A self-help version of the New Forest Parenting Programme for parents of children with attention deficit hyperactivity disorder: a qualitative study of parent views and acceptability. Child Adolesc Ment Health.

[55] Edelstein ML, Sullivan A, Becraft JL (2023). Feasibility and acceptability of a compressed caregiver training program to treat child behavior problems. Behav Modif.

[56] Uncovska M, Freitag B, Meister S, Fehring L (2023). Patient Acceptance of prescribed and fully reimbursed mHealth apps in Germany: an UTAUT2-based Online Survey Study. J Med Syst.

[57] Venkatesh V, Thong JYL, Xu X (2012). Consumer Acceptance and Use of Information Technology: extending the Unified Theory of Acceptance and Use of Technology. MIS Q.

[58] Breitenstein SM, Gross D, Christophersen R (2014). Digital delivery methods of parenting training interventions: a systematic review. Worldviews Evid Based Nurs.

[59] Nix RL, Bierman KL, McMahon RJ, Gr CPPR (2009). How attendance and quality of participation affect treatment response to parent management training. J Consult Clin Psych.

[60] Meischke H, Lozano P, Zhou C, Garrison MM, Christakis D (2011). Engagement in my child’s asthma, an interactive web-based pediatric asthma management intervention. Int J Med Informatics.

[61] Sim WH, Jorm AF, Yap MBH. The role of parent Engagement in a Web-Based Preventive Parenting Intervention for Child Mental Health in Predicting parenting, parent and child outcomes. Int J Environ Res Public Health 2022, 19(4). 10.3390/ijerph19042191 PMC8871768 35206394

