# Supplementary material for: Acceptance and utilization of web-based self-help for caregivers of children with externalizing disorders
Source: Child Adolesc Psychiatry Ment Health. 2024 Mar 25;18:40. doi: 10.1186/s13034-024-00724-0 (PMC10964538; doi:10.1186/s13034-024-00724-0)
Supplement: Supplementary file 1 — Supplementary Material 1: Child- and Parent-Related Healthcare Services Usage [file 13034_2024_724_MOESM1_ESM.docx]

**Additional file 1** Child- and Parent-Related Healthcare Services Usage

|  | never | | only in the past | | until today | |
| --- | --- | --- | --- | --- | --- | --- |
|  | *n* | *%* | *n* | *%* | *n* | *%* |
| Child-related health intervention |  |  |  |  |  |  |
| psychotherapy | 166 | 60.1% | 37 | 13.4% | 73 | 26.4% |
| occupational therapy | 73 | 26.4% | 122 | 44.2% | 81 | 29.3% |
| physiotherapy, movement therapy | 222 | 80.4% | 46 | 16.7% | 8 | 2.9% |
| pharmacological treatment | 117 | 42.4% | 12 | 4.3% | 147 | 53.3% |
| Parent-related health intervention |  |  |  |  |  |  |
| parent management training | 232 | 84.1% | 33 | 12.0% | 11 | 4.0% |
| self-help group | 253 | 91.7% | 9 | 3.3% | 14 | 5.1% |
| internet-based self-help | 264 | 95.7% | 7 | 2.5% | 5 | 1.8% |

Note: N=276 total sample size
