# Supplementary material for: Acceptance and utilization of web-based self-help for caregivers of children with externalizing disorders
Source: Child Adolesc Psychiatry Ment Health. 2024 Mar 25;18:40. doi: 10.1186/s13034-024-00724-0 (PMC10964538; doi:10.1186/s13034-024-00724-0)
Supplement: Supplementary file 2 — Supplementary Material 2: Measures of acceptance [file 13034_2024_724_MOESM2_ESM.docx]

**Additional file 5** Active and Passive Utilization

|  |  | Active utilization (tasks) *n = 276* | | | | Passive utilization (videos) *n = 276* | | | | Statistics | | | |
| --- | --- | --- | --- | --- | --- | --- | --- | --- | --- | --- | --- | --- | --- |
|  |  | M | SD | Min | Max | M | SD | Min | Max | *t* | *p* | *d* | *r* |
| Module 1 | Solving behavior problems | 53.6 | 34.78 | 0 | 100 | 44.68 | 38.90 | 0 | 100 | -4.206 | <.001 | -.25 | .46** |
| Module 2 | Positive relationship | 44.42 | 39.32 | 0 | 100 | 39.04 | 39.60 | 0 | 100 | 4.100 | <.001 | .25 | .85** |
| Module 3 | Self-care | 13.27 | 21.46 | 0 | 95.24 | 16.88 | 43.67 | 0 | 100 | -2.358 | .02 | -.14 | .68** |
| Module 4 | ADHD-What is it? | 55.54 | 46.93 | 0 | 100 | 49.73 | 42.08 | 0 | 100 | 3.057 | .002 | .18 | .75** |
| Total |  | 32.34 | 26.67 | 0 | 95.83 | 35.30 | 28.80 | 0 | 100 | -2.343 | .02 | -.14 | .72** |

Note: Utilization, i.e. completed tasks or videos in % (intensity of utilization).
