# Supplementary material for: Acceptance and utilization of web-based self-help for caregivers of children with externalizing disorders
Source: Child Adolesc Psychiatry Ment Health. 2024 Mar 25;18:40. doi: 10.1186/s13034-024-00724-0 (PMC10964538; doi:10.1186/s13034-024-00724-0)
Supplement: Supplementary file 3 — Supplementary Material 3: Content, Views and Intensity of Use (%) per Module [file 13034_2024_724_MOESM3_ESM.docx]

**Additional file 4** Correlation of Utilization Dimensions

|  | Correlation *r* of utilization parameters | | | |
| --- | --- | --- | --- | --- |
|  | Intensity (process, %) | Uptake Time (days) | Frequency (number of logins) | Duration  (minutes) |
| Uptake time^a)^ (days) | -.23** |  |  |  |
| Frequency (number of logins) | .73** | -.29** |  |  |
| Duration^b)^ (minutes) | .39** | -.10 | .43** |  |
| Support calls^c)^ (number) | .38** | -.10^d)^ | .27** | .08 |

Note: ^a)^ n=237; ^b)^ n=254, variable corrected for outlier values; ^c)^ n=141; ^d)^ n=126; *p≤.01; ** p≤.001
