# Supplementary material for: Acceptance and utilization of web-based self-help for caregivers of children with externalizing disorders
Source: Child Adolesc Psychiatry Ment Health. 2024 Mar 25;18:40. doi: 10.1186/s13034-024-00724-0 (PMC10964538; doi:10.1186/s13034-024-00724-0)
Supplement: Supplementary file 4 — Supplementary Material 4: Correlation of Utilization Dimensions [file 13034_2024_724_MOESM4_ESM.docx]

**Additional file 6** Decision Paths for CART Decision Trees

Decision paths are reported starting at the maximum (i.e. right end node) for each dependent variable, then inline leftwards.

| **Nodes** | **Decision tree predicting initial uptake of the WASH intervention** |
| --- | --- |
| 1 | If the reported value on the PNPQ (scale: positive parenting) < 3.6, the value for ADHD symptomatology < 2.7, the value for ADHD skills and reflectivity < 1.8, and the caregiver’s age (in years) ≥ 38, then caregivers were likely to take up the intervention (n=180 (65.2%) accepter/ n=14 (5%) non-accepter). |
| 2 | If the reported value on the PNPQ (scale: positive parenting) < 3.6, the value for ADHD symptomatology < 2.7, the value for ADHD skills and reflectivity < 1.8, the caregiver’s age (in years) < 38, the child’s age < 11 and the value for ADHD skills and reflectivity is ≥ 0.25, then caregivers were likely to take up the intervention with 15 % taking up and only 2 % not taking up. |
| X | Due to very small subsamples (n=5-7) and nearly 50/50 ratio for initial uptake or no initial uptake, no decision paths predicting no initial uptake are reported at this point but can be read from the presented Figure 3. |
| **Nodes** | **Decision tree predicting return after initial uptake of the WASH intervention** |
| 1 | If caregivers received ≥ 2 support calls and the reported value for ADHD symptomatology ≥ 1.3, then caregivers were more likely to return to the intervention for login (n=84 return/ n=7 no return). |
| 2 | If caregivers received ≥ 2 support calls, the reported value for ADHD symptomatology < 1.3 and the reported value for ADHD skills and reflectivity < 1.3, then caregivers were likely to return to the intervention for login (18/4). |
| 3 | If caregivers received ≥ 2 support calls, the reported value for ADHD symptomatology < 1.3 but the reported value for ADHD skills and reflectivity ≥ 1.3, then the relation of caregivers that did not return to the intervention for login changed (5/4), resulting in a tendency towards a higher risk of not returning. |
| 4 | If caregivers received < 2 support calls, the reported value for ADHD < 2.4 but ≥ 2, then caregivers were likely to return to the intervention for login (28/5). |
| 5 | If caregivers received < 2 support calls, the reported value for ADHD < 2.4 and < 2, plus oppositional behavior (ODD OPP) < 1.7 and the reported value for parental psychopathology (DASS) ≥ 0.32, then caregivers were likely to return to the intervention for login (35/7). |
| 6 | If caregivers received < 2 support calls, the reported value for ADHD < 2.4 and <2, plus oppositional behavior (ODD OPP) < 1.7 and the reported value for parental psychopathology (DASS) < 0.32, then caregivers were, as a tendency, likely to return to the intervention for login (7/2). |
| 7 | If caregivers received < 2 support calls, the reported value for ADHD < 2.4 (and < 2), plus oppositional behavior (ODD OPP) ≥ 1.7 and ADHD was <1.9 but parental psychopathology (DASS) ≥ 0.51, then caregivers were, as a tendency, likely to return to the intervention for login (8/3). |
| 8 | If caregivers received < 2 support calls, the reported value for ADHD was < 2.4 (and < 2), plus oppositional behavior (ODD OPP) ≥ 1.7 and ADHD < 1.9 but parental psychopathology (DASS) < 0.51, then the relation of caregivers that did not return to the intervention for login changed, resulting in a tendency towards a higher risk of not returning (8/3). |
| 9 | If caregivers received < 2 support calls, the reported value for ADHD < 2.4 (and < 2), plus oppositional behavior (ODD OPP) ≥ 1.7 and ADHD ≥ 1.9, then caregivers did not return to the intervention for login (9/0). |
| 10 | If caregivers received < 2 support calls, the reported value for ADHD ≥ 2.4 and positive parenting (PNPQ) < 3, then likelihood of returning to the intervention was almost 50% (3/2). |
| 11 | If caregivers received < 2 support calls, the reported value for ADHD ≥ 2.4 and positive parenting (PNPQ) ≥ 3, then caregivers did not return to the intervention for login (13/1). |
| **Nodes** | **Decision tree predicting frequency of utilization (number of logins)** |
| 1 | If number of support calls ≥ 2, the reported value for ADHD skills and reflectivity < 1, parental psychopathology (DASS) ≥ 0.6, and caregiver’s age ≥ 50, then number of logins is at maximum (13). |
| 2 | If number of support calls ≥ 2 and ADHD skills and reflectivity < 1 and DASS ≥ 0.6 and caregiver’s age < 50 and the child’s gender is female, then number of logins is 12. |
| 3 | If number of support calls ≥ 2 and ADHD skills and reflectivity < 1 and DASS ≥ 0.6 and caregiver’s age < 50 and the child’s gender is male, then number of logins is 7. |
| 4 | If number of support calls ≥ 2 and ADHD skills and reflectivity < 1 and DASS < 0.6, then number of logins is 6. |
| 4 | If number of support calls ≥ 2 and ADHD skills and reflectivity ≥ 1, then number of logins is 4. |
| 5 | If number of support calls is < 2, oppositional behavior (ODD OPP) < 2, caregivers reported being married/ in partnership with afternoon care for the child(ren), caregiver’s age ≥ 39 and ADHD symptomatology ≥ 2, then number of logins is 11. |
| 6 | If number of support calls is < 2, oppositional behavior (ODD OPP) < 2, caregivers reported being married/ in partnership with afternoon care for the child(ren), caregiver’s age ≥ 39 and ADHD symptomatology < 2, then number of logins is 6. |
| 7 | If number of support calls is < 2, oppositional behavior (ODD OPP) < 2, caregivers reported being married/ in partnership without afternoon care for the child(ren), child has never received occupational therapy, and positive parenting skills < 3, then number of logins is 9. |
| 8 | If number of support calls is < 2, oppositional behavior (ODD OPP) < 2, caregivers reported being married/ in partnership without afternoon care for the child(ren), child has never received occupational therapy, and positive parenting skills ≥ 3, then number of logins is 3. |
| 9 | If number of support calls is < 2, oppositional behavior (ODD OPP) < 2, caregivers reported being married/ in partnership without afternoon care for the child(ren) and the child receives occupational therapy (current or past), then number of logins is 3. |
| 10 | If number of support calls is < 2, oppositional behavior (ODD OPP) < 2, caregivers reported being married/ in partnership without afternoon care for the child(ren), child has never received occupational therapy, and positive parenting skills ≥ 3, then number of logins is 3. |
| 11 | If number of support calls is < 2, oppositional behavior (ODD OPP) < 2 but caregiver reported not being married / in partnership, then number of logins is 2. |
| 12 | If number of support calls is < 2, oppositional behavior (ODD OPP) ≥ 2, and caregiver reported < 4 people in their household, then number of logins is 5. |
| 13 | If number of support calls is < 2, oppositional behavior (ODD OPP) ≥ 2 and caregiver reported ≥ 4 people in their household, then number of logins is at minimum (1). |
| **Nodes** | **Decision rules for the prediction of intensity of utilization (%)** |
| 1 | If caregivers received all six support calls, had been referred to the study by their pediatrician, and reported ODD impairment ≥ 1.3, then maximum progress was processed (59%). |
| 2 | If caregivers received all six support calls, had been referred to the study by their pediatrician, and reported ODD impairment < 1.3, then 39% was processed. |
| 3 | If caregivers received all six support calls, had been referred to the study by their child and youth psychiatrist, then 36% progress was processed. |
| 4 | If caregivers received less than six support calls and caregiver’s age ≥ 47, then 38% progress was processed. |
| 5 | If caregivers received less than six support calls and caregiver’s age < 47, caregivers reported high educational attainment, and ADHD symptomatology of the child < 2.3 plus ADHD impairment ≥ 1.7, then 52% progress was processed. |
| 6 | If caregivers received less than six support calls and caregiver’s age < 47, caregivers reported high educational attainment, and ADHD symptomatology of the child < 2.3 plus ADHD impairment < 1.7, then 27% progress was processed. |
| 7 | If caregivers received less than six support calls and caregiver’s age < 47, caregivers reported high educational attainment, and ADHD symptomatology of the child ≥ 2.3, then 11% progress was processed. |
| 8 | If caregivers received less than six support calls and caregiver’s age < 47, caregivers reported low to intermediate educational attainment, and ADHD skills and reflectivity < 0.92, prosocial behavior (ODD PBS) of the child ≥ 1.4, and positive parenting skills < 3.1, then 35% progress was processed. |
| 9 | If caregivers received less than six support calls and caregiver’s age < 47, caregivers reported low to intermediate educational attainment, and ADHD skills and reflectivity < 0.92, prosocial behavior (ODD PBS) of the child ≥ 1.4 and positive parenting skills ≥ 3.1, then 18% progress was processed. |
| 10 | If caregivers received less than six support calls and caregiver’s age < 47, caregivers reported low to intermediate educational attainment, and ADHD skills and reflectivity < 0.92, prosocial behavior (ODD PBS) of the child < 1.4, then 8.4% progress was processed. |
| 11 | If caregivers received less than six support calls and caregiver’s age < 47, caregivers reported low to intermediate educational attainment, and ADHD skills and reflectivity ≥ 0.92, then 11% progress was processed. |
