# Supplementary material for: Acceptance and utilization of web-based self-help for caregivers of children with externalizing disorders
Source: Child Adolesc Psychiatry Ment Health. 2024 Mar 25;18:40. doi: 10.1186/s13034-024-00724-0 (PMC10964538; doi:10.1186/s13034-024-00724-0)
Supplement: Supplementary file 5 — Supplementary Material 5: Active and Passive Utilization [file 13034_2024_724_MOESM5_ESM.docx]

**Additional file 3** Content, Views and Intensity of Use (%) per Module

| Module   Situation | Content | Viewed^1^ | |  | Intensity of Use (%)^2^ | | | |
| --- | --- | --- | --- | --- | --- | --- | --- | --- |
|  |  |  |  |  | M | SD | Min | Max |
| Module 1 | Solving behavioral problems | 237 | (85.9%) |  | 34.73 | 21.41 | 0 | 100.00 |
| Situation 1 | Temper tantrum | 175 | (63.4%) |  | 19.99 | 26.69 | 0 | 98.28 |
| Situation 2 | Homework | 182 | (65.9%) |  | 14.11 | 22.45 | 0 | 83.67 |
| Situation 3 | Chaos in child’s room | 126 | (45.7%) |  | 8.22 | 20.08 | 0 | 100.00 |
| Situation 4 | Constant interruption | 249 | (90.2%) |  | 7.78 | 15.80 | 0 | 83.67 |
| Situation 5 | Media consumption | 124 | (44.9%) |  | 9.57 | 21.85 | 0 | 90.00 |
| Situation 6 | Sibling conflicts | 161 | (58.3%) |  | 7.07 | 17.46 | 0 | 89.80 |
| Situation 7 | Restlessness at meals | 162 | (58.7%) |  | 6.02 | 15.08 | 0 | 85.71 |
| Module 2 | Positive relationship | 237 | (85.9%) |  | 41.79 | 38.18 | 0 | 100.00 |
| Module 3 | Self-care | 237 | (85.9%) |  | 13.80 | 22.50 | 0 | 96.15 |
| Module 4 | ADHD-What is it? | 205 | (74.3%) |  | 53.20 | 42.71 | 0 | 100.00 |
| **Module 1 – 4** | **Total Utilization** | **237** | **(85.9%)** |  | **31.17** | **26.18** | **0** | **96.70** |

Note: ^1^ number of participants who have seen any of the content in this module or situation, ^2^ mean percentage of completed tasks achieved by participants within each module or situation
