# Supplementary material for: Acceptance and utilization of web-based self-help for caregivers of children with externalizing disorders
Source: Child Adolesc Psychiatry Ment Health. 2024 Mar 25;18:40. doi: 10.1186/s13034-024-00724-0 (PMC10964538; doi:10.1186/s13034-024-00724-0)
Supplement: Supplementary file 6 — Supplementary Material 6: Decision Paths for CART Decision Trees [file 13034_2024_724_MOESM6_ESM.docx]

**Additional file 2** Measures of acceptance

**Uptake Rate**i.e. used the program at least once
85.9% (n= 237)

T1 participants

N= 276

3 months of intervention

*Objective measures*

T2 participants
N= 187

N=170

**Return Rate**71.4% (n= 197)

“have **used** the program**”**

91.4% (n= 171)

“would (rather) **recommend** the program to a friend”
95.9%(n= 163)

*subjective measures*
